# TPX2 Serves as a Cancer Susceptibility Gene and Is Closely Associated with the Poor Prognosis of Endometrial Cancer

**DOI:** 10.1155/2022/5401106

**Published:** 2022-03-16

**Authors:** Jun Wang, Hua Zheng, Hui He, Shuying Meng, Yatian Han, Zhe Su, Hanbing Yan, Yanan Zhang

**Affiliations:** ^1^Department of Obstetrics and Gynecology, Benxi Central Hospital of China Medical University, Benxi, Liaoning 117022, China; ^2^Clinical Laboratory Department, Benxi Central Hospital of China Medical University, Benxi, Liaoning 117022, China; ^3^Key Laboratory of Obstetrics and Gynecology of Higher Education of Liaoning Province, Shenyang, Liaoning 110000, China; ^4^Key Laboratory of Maternal-Fetal Medicine of Liaoning Province, Shenyang, Liaoning 110000, China; ^5^Department of Oncology, The Affiliated Benxi Jinshan Hospital of Dalian Medical University, Benxi, Liaoning 117022, China; ^6^Department of Pathology, Benxi Central Hospital of China Medical University, Benxi, Liaoning 117022, China

## Abstract

**Background:**

Endometrial cancer (EC) is a common tumor of the genital tract that affects the female reproductive system but with only limited treatment options. We aimed to discover new prognostic biomarkers for EC.

**Methods:**

We used mRNA-seq data to detect differentially expressed genes (DEGs) between EC and control tissues. Detailed clinicopathological information was collected, and changes in the mRNA and protein levels of hub DEGs were analyzed in EC. Copy number variation (CNV) was also evaluated for its association with the pathogenesis of EC. Gene set enrichment analysis (GSEA) was conducted to enrich significant pathways driven by the hub genes. Cox regression analysis was used to select variables to create a nomogram. The nomogram was calibrated by applying the concordance index (C-index), and net benefits of the nomogram at different threshold probabilities were quantified using decision curve analysis (DCA).

**Results:**

Differential expression analysis identified 24 DEGs as potential risk factors for EC. Survival analysis revealed that TPX2 expression was related to worsening overall survival in patients with advanced EC. A high CNV was associated with the overexpression of TPX2; this suggested that modifications in the cell-cycle pathway might be crucial in the advancement of EC. Moreover, an individualized nomogram was developed for TPX2 incorporating clinical factors; this was also evaluated for its ability to predict EC. Calibration and DCA analyses confirmed the robustness and clinical usefulness of the nomogram.

**Conclusion:**

We offer novel insights into the pathogenesis and molecular mechanisms of EC. The overexpression of TPX2 was related to a poorer prognosis and could serve as a biomarker for predicting prognostic outcomes in EC patients.

## 1. Introduction

Endometrial cancer (EC) is the sixth most frequent malignancy among women worldwide [1]. In the early stages of the disease, the 5-year relative survival rate is more than 95%. However, the survival rates of patients in the advanced stages range from 20% to 40% [2]. Although surgery, chemotherapy, and radiotherapy can be used to treat EC patients, there is still a lack of effective therapeutic targets. Thus, there is a clear need of new diagnostic and prognostic biomarkers for new treatment strategies for patients with EC.

The targeting protein for Xenopus kinesin-like protein 2 (TPX2) is a key factor that ensures the correct assembly of the mitotic spindle and is located on chromosome 20q11.1 in humans [3]. Although TPX2 is closely associated with the spindle pole during mitosis, it disappears after the completion of cytokinesis [4,5]. Similar to other proteins that regulate mitosis, TPX2 is overexpressed in a range of different cancers and is generally associated with a poor prognosis. The increased protein expression of TPX2 reportedly improves the proliferative, invasive, and migratory abilities of colorectal and cervical cancers [6,7]. However, the downregulation of TPX2 in hepatocellular tumors could suppress proliferative, invasive, and migratory abilities *via* the PI3K/AKT/mTOR pathway [8]. However, there is lack of studies on the exact role of TPX2 in the development of EC.

Here, we explored the effects of TPX2 expression on overall survival (OS) in EC patients and further investigated the molecular mechanisms underlying its differential expression. Following univariate and multivariate COX regression analyses, we constructed a TPX2 nomogram with independent prognostic factors, effectively predicting 1-, 3-, and 5-year overall survival (OS) in patients with EC. In summary, TPX2 is associated with the pathogenesis of EC and serves as a marker for the prognostic evaluation of EC.

## 2. Materials and Methods

### 2.1. Patients and Specimens

We retrieved messenger RNA expression datasets (GSE63678 and GSE17025) from the Gene Expression Omnibus (GEO) database (https://www.ncbi.nlm.nih.gov/gds/) to screen for candidate genes. GSE63678 featured 7 EC and 5 normal endometrial tissues. GSE17025 contained 91 EC and 12 normal endometrial tissues. Subsequently, the clinical data and expression profiles of the EC patients (*n* = 575; EC, 552; normal endometrial tissue, 23) were retrieved from The Cancer Genome Atlas (TCGA) database (https://cancergenome.nih.gov) so that we could evaluate prognostic markers.

Endometrial samples were collected from 68 surgical patients who underwent surgery at the Department of Gynecology of Benxi Central Hospital between March 2020 and March 2021. The 68 samples comprised 25 endometrial carcinomas, 5 serous carcinomas, 1 clear cell carcinoma, and 37 normal endometrial tissues (proliferative endometrial specimens, 15; secretory endometrial specimens, 5; and atrophic endometrial specimens, 17). The median ages of patients with malignant and normal endometrial samples were 57.4 (41–75) and 54.9 (31–80) years, respectively. The patients had not received radiotherapy, chemotherapy, or hormone therapy, and all patients provided written and informed consent. Experiments were approved by the Clinical Research Ethics Committee of the Benxi Central Hospital of China Medical University (23/04/2020; reference: 20200309-1).

### 2.2. Screening for Differentially Expressed Genes (DEGs)

The GEO2R tool (https://www.ncbi.nlm.nih.gov/geo/geo2r/) was used to screen for DEGs in both the GSE63678 and GSE17025 datasets. Differential expression analyses of mRNAs in EC and control tissues from the TCGA datasets were conducted using the Bioconductor Linear Model for Microarray Analysis (LIMMA) R package. Genes with a |log2(fold-change)| of >2.0 and *p* < 0.05 were considered as potential DEGs and were subjected to further analysis. The Venny v2.1 web-based tool (https://bioinfogp.cnb.csic.es/tools/venny/index.html) was utilized to identify candidate DEGs among the three datasets.

### 2.3. Survival Analysis and Expression Validation of Hub DEGs

The TCGA-EC samples (*n* = 552) were assigned to low- and high-expression groups based on the cutoff point (the median expression value of the DEGs). Kaplan–Meier survival curves were plotted using the survival R package. To further evaluate whether the candidate DEGs can serve as prognostic factors for EC, we performed univariate and multivariate Cox regression analyses. The mRNA expression levels of each DEG were verified with Oncomine (https://www.oncomine.org/) microarray data sheets according to the fold-change and threshold *p* value of 2 and 1 × 10^−4^, respectively.

### 2.4. Western Blotting

Sixty-eight endometrial specimens were used to screen the proteins encoded by hub genes by western blotting with a monoclonal TPX2 antibody (1 : 500, EPR23180-4; Abcam, USA). *β*-Actin was employed as an internal control.

### 2.5. Histology and Immunostaining

Immunohistochemical staining was performed using tissue microarrays (TMAs; EMC1351; Superbiotek, Shanghai, China) containing EC and paracancerous tissue samples (*n* = 17) for differential expression analysis of hub genes. The experimental methodology used here was previously described by Lei et al. [9].

### 2.6. CNV Data Analysis

The Affymetrix SNP 6.0 platform on the Genomic Data Commons (GDC) Cancer Browser (https://portal.gdc.cancer.gov/) was used to retrieve TCGA-EC copy number variation (CNV) data. Genes that were fully located in the significantly aberrant CNV regions were then identified by alignment with the genome.

### 2.7. Gene Set Enrichment Analysis (GSEA)

GSEA was conducted to identify enriched genes related to high-expression levels of the hub genes in EC patients. In particular, we used the GSEA preranked function; the genes were sorted according to fold-change, the number of permutations was fixed at 10,000, and the size of a gene set was limited to 2000 genes.

### 2.8. Prognostic Value Analysis

Prognostic information and clinicopathological data, such as age, tumor grade, Fédération Internationale de Gynécologie et d'Obstétrique (FIGO) stage, and the histological types of 552 patients were obtained from the TCGA-EC cohort. The TCGA-EC samples (*n* = 552) were randomly divided into a training group (*n* = 276) and a verification group (*n* = 276). There were no significant differences between the training and verification groups in terms of age, tumor grade, FIGO stage, or histological type (Supplementary Table 1). Using the combination group (*n* = 552), we performed survival analysis to investigate the relationship between the mRNA expression of each hub gene and its corresponding CNV and clinical outcomes. Next, univariate and multivariate Cox regression analyses were conducted to identify whether the hub gene was independent risk factor.

Based on the independent prognostic factors identified in the final multivariate Cox regression analysis, we used a nomogram to predict OS among EC patients in the training group. The nomogram was visually assessed using a calibration plot that compared the predicted and actual survival probabilities of EC patients. The prognostic performance of the nomogram was determined by the area under the ROC curve (AUC), which can range from 0.5 (no discrimination) to 1 (perfect discrimination). Furthermore, we used decision curve analysis (DCA) to compare a nomogram that included all independent prognostic factors with only one independent prognostic factor [10]. The DCA was used to calculate the clinical net benefit of each model compared to all or no strategies. The best model was the one with the highest net benefit as calculated.

### 2.9. Statistical Analysis

Perl scripting tool v5.26.3, R software v3.5.3, and R Studio v1.1.463 were employed for statistical analysis. The Student's *t*-test was applied to compare the mean values between the groups, and all data were checked for normal distribution and homogeneity of variance using the Shapiro–Wilk test and the Levene test, respectively. Fisher's exact test was employed to determine the association between hub gene expression and clinicopathological characteristics in TMA-EC samples. The optimal cutoff age for patients with TCGA-EC was calculated with X-tile software v3.6.1 according to the survival status [11]. The effect of hub genes on OS in EC patients was assessed using Kaplan–Meier survival curves and the log-rank test. Significance level was set at *p* < 0.05.

## 3. Results

### 3.1. Screening for Hub Genes

**Figure 1:**
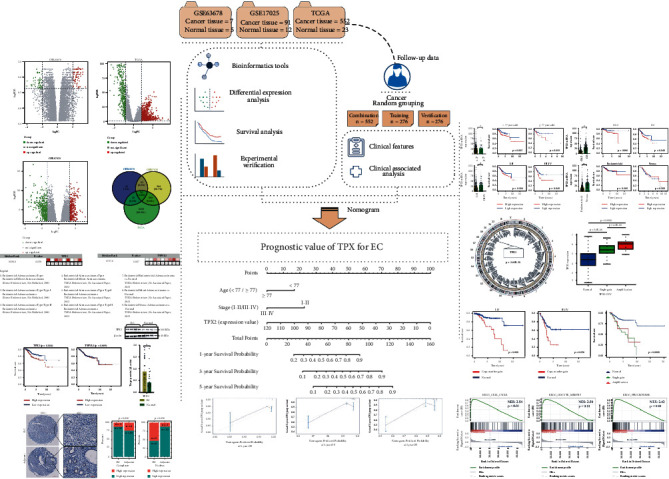
Flow diagram depicting the flow of work in this study. EC: endometrial cancer; CNV: copy number variation; GSEA: gene set enrichment analysis; DEG: differentially expressed gene.

**Figure 2:**
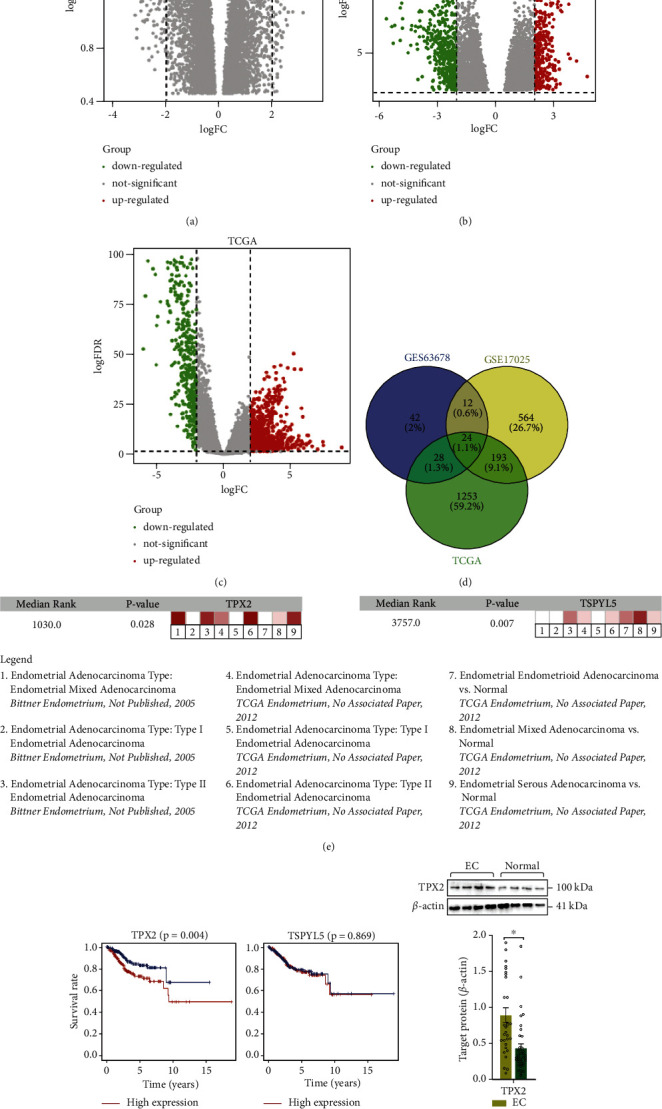
DEG screening in EC patients. (a–c) Volcano plots showing the difference in mRNA expression between EC and normal endometrial tissues. (d) Venn diagram showing overlap among candidate mRNAs with a |log2 (fold -change)| of >2.0 in EC tissue compared with those in normal endometrial tissue. (e) The mRNA level of each candidate DEG was validated with the oncomine microarray database. (f) Comparison of the survival curves between low- and high-expression groups based on the median expression value of candidate differentially expressed genes. (g) Immunoblot showing the prognostic signature associated with TPX2 protein and quantitative data. ^*∗*^*p* < 0.05 vs. normal group. EC: endometrial cancer; FC: fold change; FDR: false discovery rate.

Figure 1 shows a flowchart depicting the flow of work in this study. The GSE63678 dataset contained 70 upregulated and 50 downregulated DEGs (Figure 2(a)), while the GSE17025 dataset contained 580 upregulated and 350 downregulated DEGs (Figure 2(b)). In the TCGA Uterine Corpus Endometrial Carcinoma (UCEC) dataset, we identified 1087 upregulated genes and 411 downregulated genes (Figure 2(c)).  Figure 2(d) shows a Venn diagram depicting the 24 candidate genes that were differentially expressed (see also Supplementary Table 2).

### 3.2. Identification of the Hub DEGs in EC Patients

**Figure 3:**
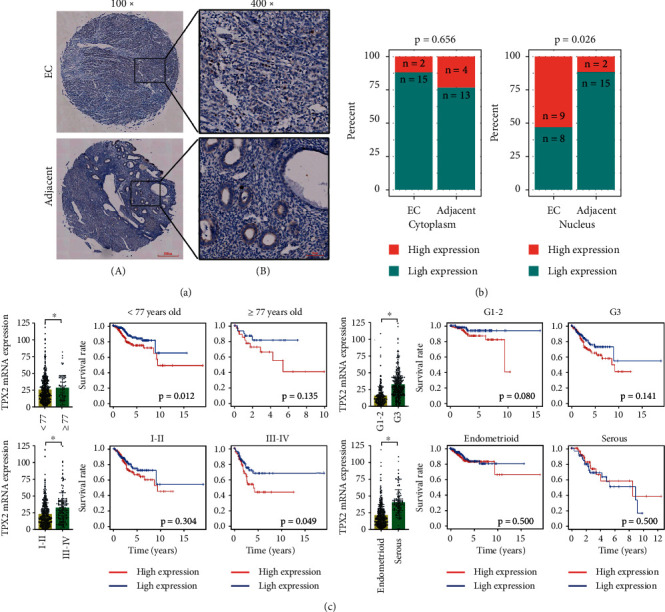
Determination of the tissue distribution of TPX2. (a) IHC images showing the cellular localization of TPX2 in cancerous and adjacent tissue sections from EC patients. Scale bar: 200 *μ*m, 100x magnification (A); scale bar: 50 *μ*m, 400x magnification (B). (b) Comparison of TPX2 distribution between cancerous and adjacent tissues. (c) The mRNA expression and effect of TPX2 on OS in EC patients stratified by age, tumor grade, FIGO stage, and histological type. ^*∗*^*p* < 0.05 indicates significant subgroup difference based on the unpaired student's t-test.

Univariate regression analysis indicated that 16 candidate DEGs were risk-associated genes for EC. Multivariate regression analysis demonstrated that TPX2 (hazard ratio (HR): 1.035; 95% confidence interval (CI): 1.025-1.045; *p* = 6.07*E* − 13) and testis-specific Y-encoded-like protein 5 (TSPYL5) (HR: 1.034; 95% CI: 1.007–1.061; *p*=0.013) were independent risk factors of EC pathogenesis (Table 1). Oncomine coexpression analysis revealed that the expression patterns of these two hub genes were in good agreement with our initial analysis (Figure 2(e)). Furthermore, survival analysis showed that only the overexpression of TPX2 could contribute to the prognosis of EC patients (Figure 2(f)). Moreover, the levels of TPX2, as confirmed by western blotting, were significantly higher in EC tissue than in normal endometrial tissue (0.893 ± 0.102 vs. 0.438 ± 0.062, *p* < 0.001) (Figure 2(g)). These findings are consistent with the mRNA data. In addition, IHC showed that TPX2 (1 : 250, EPR23180-4; Abcam) was primarily localized in the cytoplasm and nucleus (Figure 3(a)). Compared with the adjacent tissues, the levels of TPX2 were considerably higher in the nucleus in EC tissues (Figure 3(b)).

### 3.3. Correlation between TPX2 Expression and Clinicopathologic Features

Next, we used TCGA-EC mRNA expression data relating to TPX2 and clinicopathologic features to perform differential expression analysis and survival analysis. These parameters included age (Supplementary Figure 1), tumor grade, FIGO stage, histological types, and survival status. Although the mRNA levels of TPX2 in EC patients with G3 (mean ± SEM: 33.780 ± 1.084 vs. 15.490 ± 0.799), stage III-IV (33.290 ± 1.787 vs. 23.690 ± 0.862), and serous disease (40.970 ± 1.769 vs. 21.870 ± 0.815) were higher than those in the control group (all *p* < 0.001), there was only an effect on OS when the mRNA expression of TPX2 was increased in patients with advanced stage III-IV EC (Figure 3(c)).

### 3.4. CNV Analysis of TPX2 in EC Patients

**Figure 4:**
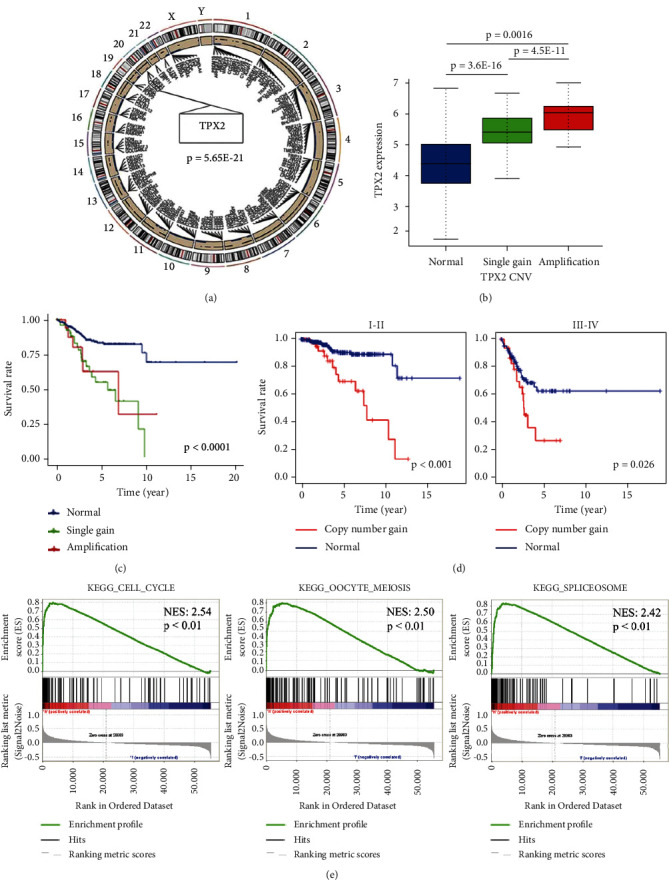
CNV analysis and GSEA of TPX2 in EC patients. (a) The circos of the human genome illustrating the chromosomal structure and localization of TPX2 CNVs in endometrial carcinoma. The outer most layer denotesthe chromosome model while the next layer indicates CNVs (blue dot: deletion; black dot: amplification). (b) TPX2 copy number gain and the corresponding expression levels of TPX2 mRNA (blue: copy number normal; green: single gain; red: amplification). (c) The effect of TPX2 copy number gain (including single gain and amplification) on OS in EC patients. (d) The effect of TPX2 copy number gain on OS of EC patients based on the FIGO stage. (e) Gene sets enriched in the group with high TPX2 expression. NES: normalized enrichment score.

CNV mapping onto the entire genome revealed that the TPX2 segment was considerably amplified in the endometrial tumor group when compared with that in the normal endometrial group; these findings were consistent with the TPX2 mRNA expression data (Figures 4(a) and 4(b)). Moreover, the copy number amplification of TPX2 in EC tissue was also related to a poor prognosis, especially with FIGO stage (Figures 4(c) and 4(d), Supplementary Figure 2).

### 3.5. GSEA Identification of TPX2-Related Signaling Pathways

GSEA was performed by comparing high- and low-TPX2 expression groups to investigate the potential function of TPX2 in EC. The enriched gene sets with a false discovery rate of <0.25 and *p* < 0.05 were considered statistically significant. As shown in Figure 4(e), the top three enriched phenotypes in the high-TPX2 expression group were “cell cycle,” “oocyte meiosis,” and “spliceosome,” while the pathways enriched in the TPX2-low expression group were “alpha-linolenic acid metabolism,” “complement and coagulation cascades,” and “linoleic acid metabolism” (Supplementary Figure 3).

### 3.6. OS Prediction and Evaluation

**Figure 5:**
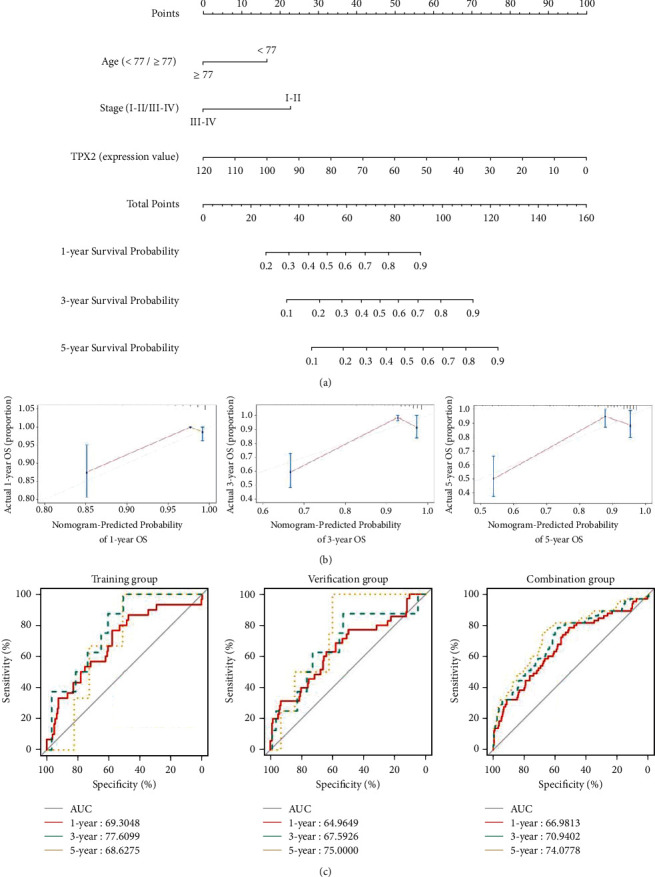
Analysis of a nomogram for predicting OS in EC patients. (a) Nomogram of three independent risk factors (age, FIGO stage, and TPX2); the scores for each variable were added to obtain the total score for predicting the 1-, 3- and 5-year OS of EC patients. (b) Calibration plots for the nomogram of the training group predicting 1- (*n* = 217), 3- (*n* = 99) and 5-year (*n* = 54). The reference line represents a perfect match between the predicted and actual survival probabilities. (c) AUC of the ROC curve verifying the prognostic accuracy of the nomogram for predicting 1-, 3- and 5-year OS. OS: overall survival; EC: endometrial cancer; AUC: area under the curve; ROC: receiver operating characteristic.

**Figure 6:**
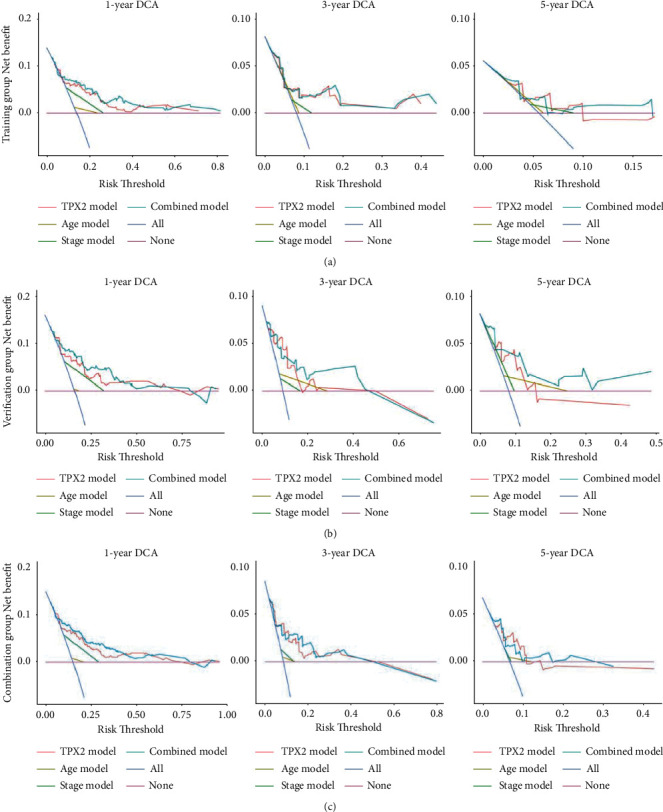
DCA curves of the nomogram. A comparison of the DCA curves for 1-, 3- and 5-year overall survival in EC patients among the training group, verification group and combination groups, respectively. The none plot represents the assumption that no patient presented 1-, 3- or 5-year survival; whereas all plot represents the assumption that all patients presented 1-, 3- or 5-year survival at a specific threshold probability. The *x*-axis represents the threshold probabilities, and the *y*-axis shows the net benefit. DCA: decision curve analysis; EC: endometrial cancer.

To further evaluate whether TPX2 can serve as a prognostic factor, we performed univariate and multivariate Cox regression analyses to compare EC patients with high and low levels of TPX2 expression. Apart from TPX2, we also tested the effect of other covariates, such as age, tumor grade, FIGO stage, and histological type. Multivariate Cox regression analysis indicated that TPX2 (HR: 1.033; 95%CI: 1.023–1.043; *p* < 0.001), age (HR: 2.114; 95% CI: 1.211–3.689; *p* < 0.01), and FIGO stage (HR: 2.706; 95% CI: 1.726–4.240; *p* < 0.001) were independent risk factors and had better prognostic value (Table 2). Subsequently, we constructed a nomogram to predict the 1-, 3-, and 5-year OS of EC patients using TPX2, age, and FIGO stage (Figures 5(a) and 5(b), Supplementary Figure 4). The C-index for the training group, verification group, and combination group was 0.838 (95% CI: 0.763–0.897), 0.779 (95% CI 0.702–0.856), and 0.803 (95% CI 0.753–0.856), respectively. The AUCs for 1-, 3-, and 5-year OS were 0.693, 0.776, and 0.686; 0.650, 0.676, and 0.750; and 0.670, 0.709, and 0.741 for the training group, verification group, and combination group, respectively (Figure 5(c)). Compared with a nomogram that only included the TPX2, age, or FIGO stage, the combined model exhibited the best net benefit for 1-, 3-, and 5-year OS (especially for the net benefit for 5-year OS in EC patients), although TPX2 could independently increase the net benefit for the 1-, 3-, and 5-year OS of EC (Figures 6(a)–6(c)).

## 4. Discussion

EC is the most frequent gynecological malignancy and the sixth most frequently diagnosed form of cancer globally, with more than 417,000 new cases and 97,000 deaths in 2020 [12]. EC can be divided into two pathogenetic types according to the occurrence of hyperlipidemia, obesity, and hyperestrogenism [13]. Histopathologically, type I EC is characterized by good endometrial differentiation, progesterone sensitivity, and a better prognosis; endometrioid carcinoma is the most common histological type. In contrast, type II EC is characterized by poor differentiation, progesterone resistance, and a worse prognosis; serous carcinoma is the most common histological type [14]. The early detection of EC is associated with favorable OS and excellent quality of life postsurgery, whereas patients with an advanced disease lack effective treatment. Although adjuvant chemotherapy, radiotherapy, and targeted therapy have significantly prolonged the OS of patients with advanced EC, the prognosis remains poor [15,16]. Furthermore, current diagnostic biomarkers fail to predict the progression of EC. Thus, there is a need to identify reliable biomarkers for the early diagnosis and prognosis prediction of EC.

Owing to the genetic heterogeneity of cancer, we need comprehensive data to identify biomarkers to achieve precision diagnosis and treatment. We integrated two GEO-EC datasets and TCGA-EC mRNA-seq data for DEG screening. Twenty-four genes were differentially expressed in all three databases. Univariate and multivariate COX regression analyses revealed that TPX2 and TSPYL5 were independent risk factors for EC. We investigated the effects of these risk factors on the OS of EC patients in the TCGA-EC cohort. These findings revealed that only the overexpression of TPX2 could contribute to poor prognosis, thus suggesting that TPX2 may represent a hub gene for the accurate prediction of prognostic outcomes in EC patients.

We exploited CNV data from TCGA datasets to compare differences between EC and normal endometrial tissues with respect to TPX2 CNV fragments. The copy number of TPX2 fragments was considerably higher in EC patients when compared with that in normal controls; we also investigated the corresponding TPX2 mRNA expression data. Recent technological advances in DNA sequencing have enabled a more detailed understanding of the molecular changes that define gynecological tumors [17], and the association between TPX2 overexpression and copy number amplification has been reported in malignant tumors of the ovaries and cervix [18,19]. Here, we revealed a correlation between TPX2 overexpression and copy number amplification in EC patients, especially those with FIGO stages III and IV; these stages are strongly associated with a poor prognosis. These data indicate that TPX2 copy number gains may play a key role in carcinogenesis and disease progression.

Although the precise role of TPX2 in EC tumorigenesis has yet to be fully elucidated, there is evidence to suggest that TPX2 plays a role in the pathogenesis of various cancers *via* immune infiltration, the AKT pathway, and by regulating TP53 activity [20–22]. One of the most significant findings to emerge from the present study is that the distribution and expression patterns of TPX2 in the nuclei of EC cells were considerably higher than those in the adjacent tissues. Furthermore, GSEA demonstrated significant enrichment of genes related to the cell cycle within the group of patients with high TPX2 expression levels. TPX2 is a mitotic regulator that participates in the microtubular formation of spindles for chromosomal division during the cell cycle [23–25]. Degeneration of the cell cycle is a common occurrence in human cancer; several reports have shown that TPX2, along with other mitotic regulators, and particularly Aurora-A, synergistically promote chromosomal instability in tumor cells by impairing appropriate spindle assembly and by inducing mitotic errors [26–29]. Furthermore, the excessive expression of TPX2 also affects the microtubule cytoskeleton in a manner that is independent of Aurora-A binding; this can alter the structure and distribution of organelles in retinal pigment epithelial cells [30]. The aforementioned pathways may also play functional roles in the pathogenesis of EC and genetic predisposition; however, additional investigations are needed to verify this hypothesis.

In addition, we focused on the integration of TPX2 and clinicopathological factors to predict a poorer prognosis in patients with EC. Although the aforementioned risk factors are closely related to a poor prognosis in EC, none of these factors can be used alone to predict the prognosis of EC. In this study, we demonstrated that TPX2, age, and FIGO stage had a better prognostic value for EC. We developed a novel nomogram and found that TPX2 plays an important role in predicting the 1-, 3-, and 5-year OS rates of EC patients.

This study has some limitations that need to be considered. The single nucleotide polymorphisms (SNPs) in TPX2 that confer increased susceptibility to EC need to be analyzed further. Our research group has been collecting serum samples from EC patients admitted to the Obstetrics and Gynecology Department of Benxi Central Hospital so that we can screen for high-risk SNPs. In addition, more cytological studies are warranted to investigate the roles of TPX2 in the pathogenesis of EC and to verify the association between TPX2 and EC progression.

## 5. Conclusion

In summary, TPX2 overexpression was considerably associated with a poorer prognosis in patients with EC. CNV alterations in TPX2 might be a potential mechanism for its overexpression during the development and progression of EC. TPX2 can serve as a prognostic biomarker for predicting OS in EC patients and may facilitate the development of novel gene-targeted therapy for this disease.

## Figures and Tables

**Table 1:** Univariate/multivariate cox regression analysis for hub genes.

Gene	Univariate analysis			Multivariate analysis		
	HR	95% CI	*p* value	HR	95% CI	*p* value
TPX2	1.032	1.025–1.038	1.96*E* − 20	1.035	1.025–1.045	6.07*E* − 13
TSPYL5	1.043	1.024–1.063	6.20*E* − 06	1.034	1.007–1.061	0.013
CENPF	1.060	1.038–1.082	2.24*E* − 08	1.044	0.975–1.117	0.217
ASPM	1.193	1.119–1.273	7.84*E* − 08	0.921	0.744–1.140	0.450
DLGAP5	1.082	1.050–1.116	3.54*E* − 07	1.035	0.965–0.110	0.340
CDC20	1.009	1.004–1.013	1.13*E* − 04	0.998	0.990–1.006	0.680
KIF20A	1.036	1.017–1.056	1.87*E* − 04	0.982	0.945–1.021	0.357
NCAPG2	1.120	1.054–1.190	2.55*E* − 04	1.020	0.898–1.160	0.758
CCNB2	1.026	1.009–1.058	2.16*E* − 03	1.000	0.964–1.038	0.989
PBK	1.035	1.012–1.058	2.33*E* − 03	0.992	0.952–1.035	0.726
PTTG1	1.012	1.003–1.020	5.23*E* − 03	1.001	0.985–1.021	0.922
MELK	1.033	1.009–1.058	6.09*E* − 03	0.947	0.873–1.027	0.186
ARMCX1	1.040	1.011–1.070	6.69*E* − 03	0.984	0.939–1.032	0.509
ECT2	1.016	1.003–1.028	0.012	0.992	0.955–1.032	0.701
CEP55	1.033	1.006–1.060	0.015	1.011	0.963–1.062	0.647
BIRC5	1.005	1.000–1.010	0.039	0.997	0.987–1.007	0.610
CDO1	1.050	0.998–1.106	0.060	0.995	0.930–1.065	0.885
SFN	1.000	0.998–1.000	0.119	1.003	0.999–1.001	0.864
RRM2	1.009	0.997–1.021	0.138	1.003	0.985–1.021	0.770
TOP2A	1.002	0.999–1.005	0.164	1.000	0.992–1.008	0.977
ENPP2	1.010	0.990–1.030	0.321	1.011	0.988–1.035	0.350
MMP12	0.996	0.984–1.009	0.595	0.995	0.981–1.009	0.485
CYP1B1	1.003	0.984–1.023	0.737	1.004	0.980–1.028	0.755
TCEAL2	1.003	0.983–1.024	0.743	0.994	0.970–1.020	0.662

HR: hazard ratio; CI: confidence interval.

**Table 2:** Univariate/multivariate cox regression analysis for independent prognostic factors.

Variables	Univariate analysis			Multivariate analysis		
	HR	95% CI	*p* value	HR	95% CI	*p* value
*Age*						
＜77/≥77	1.991	1.159–3.421	0.013	2.114	1.211–3.689	8.45*E* – 03

*Grade*						
G1-2/G3	3.715	2.162–6.381	2.00*E* – 06	1.310	0.699–2.455	0.400

*FIGO stage*						
I-II/III-IV	4.174	2.743–6.351	2.50*E* – 11	2.706	1.726–4.240	1.41*E* – 05

*Histological types*						
Endometrioid/serous	3.426	2.259–5.195	6.84*E* – 09	1.127	0.688–1.847	0.635

*TPX2*						
low/high	1.040	1.032–1.047	2.50*E* – 24	1.033	1.023–1.043	3.52*E* – 11

HR: hazard ratio; CI: confidence interval; FIGO: fédération internationale de gynécologie et d'obstétrique.

## Data Availability

The datasets used and/or analyzed in this study are available from the corresponding author upon reasonable request.

## Supplementary Materials

Supplementary Materials Supplementary Table 1: clinical features of EC patients in the training and verification groups. Supplementary Table 2: differentially expressed genes. Supplementary Figure 1: Kaplan–Meier survival curves and X-tile plots demonstrated that the optimal cutoff age was 77 years. Supplementary Figure 2: the effect of TPX2 copy number gain on OS in EC patients stratified by age (A), tumor grade (B), and histological type (C). OS: overall survival; EC: endometrial cancer. Supplementary Figure 3: the gene sets that showed enrichment in the group with low levels of TPX2 expression. Supplementary Figure 4: calibration plots of the nomogram. (A) Calibration plots for the nomogram created from the verification group predicting 1-year OS (*n* = 218), 3-year OS (*n* = 89), and 5-year OS (*n* = 49). (B) Calibration plots for the nomogram for the combination group predicting 1-year OS (*n* = 435), 3-year OS (*n* = 188), and 5-year OS (*n* = 103). The reference line represents a perfect match between the predicted and actual survival probabilities. OS: overall survival.
