# Supplementary material for: TPX2 Serves as a Cancer Susceptibility Gene and Is Closely Associated with the Poor Prognosis of Endometrial Cancer
Source: Genet Res (Camb). 2022 Mar 16;2022:5401106. doi: 10.1155/2022/5401106 (PMC8942693; doi:10.1155/2022/5401106)
Supplement: Supplementary Materials — Supplementary Table 1: clinical features of EC patients in the training and verification groups. Supplementary Table 2: differentially expressed genes. Supplementary Figure 1: Kaplan–Meier survival curves and X-tile plots demonstrated that the optimal cutoff age was 77 years. Supplementary Figure 2: the effect of TPX2 copy number gain on OS in EC patients stratified by age (A), tumor grade (B), and histological type (C). OS: overall survival; EC: endometrial cancer. Supplementary Figure 3: the gene sets that showed enrichment in the group with low levels of TPX2 expression. Supplementary Figure 4: calibration plots of the nomogram. (A) Calibration plots for the nomogram created from the verification group predicting 1-year OS (n = 218), 3-year OS (n = 89), and 5-year OS (n = 49). (B) Calibration plots for the nomogram for the combination group predicting 1-year OS (n = 435), 3-year OS (n = 188), and 5-year OS (n = 103). The reference line represents a perfect match between the predicted and actual survival probabilities. OS: overall survival. [file 5401106.f1.zip › 5401106.f1/Supplementary Table 1.docx]

**Supplementary Table 1. Clinical features of EC patients in the training and verification groups.**

| **Feature** | **Training group**  **(n = 276)** | **Verification group**  **(n = 276)** | ***p*-value** |
| --- | --- | --- | --- |
| ***Age*** |  |  |  |
| **<** 77 | 229 | 223 | 0.581 |
| **≥** 77 | 47 | 53 |  |
| ***Grade*** |  |  |  |
| G1–G2 | 116 | 100 | 0.174 |
| G3 | 149 | 155 |  |
| Unknown | 11 | 21 |  |
| ***FIGO stage*** |  |  |  |
| I–II | 184 | 188 | 0.271 |
| III–IV | 74 | 69 |  |
| Unknown | 18 | 29 |  |
| ***Histological type*** |  |  |  |
| Endometrioid | 199 | 212 | 0.391 |
| Serous | 48 | 54 |  |
| Unknown | 19 | 10 |  |

*EC: cervical cancer; FIGO: Fédération Internationale de Gynécologie et d’Obstétrique*
