# Supplementary material for: TPX2 Serves as a Cancer Susceptibility Gene and Is Closely Associated with the Poor Prognosis of Endometrial Cancer
Source: Genet Res (Camb). 2022 Mar 16;2022:5401106. doi: 10.1155/2022/5401106 (PMC8942693; doi:10.1155/2022/5401106)
Supplement: Supplementary Materials — Supplementary Table 1: clinical features of EC patients in the training and verification groups. Supplementary Table 2: differentially expressed genes. Supplementary Figure 1: Kaplan–Meier survival curves and X-tile plots demonstrated that the optimal cutoff age was 77 years. Supplementary Figure 2: the effect of TPX2 copy number gain on OS in EC patients stratified by age (A), tumor grade (B), and histological type (C). OS: overall survival; EC: endometrial cancer. Supplementary Figure 3: the gene sets that showed enrichment in the group with low levels of TPX2 expression. Supplementary Figure 4: calibration plots of the nomogram. (A) Calibration plots for the nomogram created from the verification group predicting 1-year OS (n = 218), 3-year OS (n = 89), and 5-year OS (n = 49). (B) Calibration plots for the nomogram for the combination group predicting 1-year OS (n = 435), 3-year OS (n = 188), and 5-year OS (n = 103). The reference line represents a perfect match between the predicted and actual survival probabilities. OS: overall survival. [file 5401106.f1.zip › 5401106.f1/Supplementary Table 2.docx]

**Supplementary Table 2. Differentially expressed genes.**

| **Gene** | **GSE63678** | | **GSE17025** | | **TCGA-EC** | |
| --- | --- | --- | --- | --- | --- | --- |
|  | **Log FC** | ***P*-Value** | **Log FC** | ***P*-Value** | **Log FC** | ***P*-Value** |
| *TCEAL2* | -2.939 | 9.25E-04 | - 2.742 | 2.02E-05 | -2.688 | 3.02E-13 |
| *ENPP2* | -2.742 | 3.27E-05 | - 2.297 | 2.24E-06 | -2.380 | 2.69E-28 |
| *ARMCX1* | -2.558 | 4.45E-04 | - 2.437 | 3.89E-08 | -2.347 | 9.45E-30 |
| *TSPYL5* | -2.404 | 7.45E-04 | - 2.154 | 1.15E-07 | -2.187 | 2.00E-12 |
| *CDO1* | -2.397 | 9.02E-05 | - 2.908 | 1.79E-10 | -2.521 | 2.21E-15 |
| *CYP1B1* | -2.331 | 2.18E-03 | -2.390 | 4.72E-08 | -3.109 | 1.20E-41 |
| *NCAPG* | 2.025 | 7.01E-05 | 2.990 | 1.13E-10 | 3.595 | 2.57E-23 |
| *CENPF* | 2.200 | 3.78E-05 | 2.327 | 1.82E-12 | 3.800 | 1.48E-29 |
| *TPX2* | 2.302 | 3.55E-04 | 2.992 | 7.09E-13 | 4.446 | 4.57E-40 |
| *KIF20A* | 2.345 | 2.74E-05 | 2.410 | 3.08E-11 | 4.163 | 2.22E-36 |
| *CDC20* | 2.454 | 7.69E-05 | 2.463 | 5.26E-13 | 3.149 | 3.34E-05 |
| *DLGAP5* | 2.458 | 1.06E-04 | 3.198 | 8.34E-12 | 4.064 | 1.86E-28 |
| *TOP2A* | 2.468 | 3.26E-05 | 3.526 | 1.39E-14 | 4.021 | 3.43E-35 |
| *ASPM* | 2.528 | 7.10E-05 | 3.761 | 4.15E-12 | 3.388 | 1.22E-15 |
| *ECT2* | 2.550 | 5.55E-05 | 2.632 | 8.65E-07 | 2.665 | 4.28E-19 |
| *BIRC5* | 2.596 | 3.77E-05 | 2.357 | 3.54E-09 | 4.577 | 1.19E-34 |
| *MELK* | 2.610 | 1.24E-05 | 3.472 | 2.18E-13 | 4.249 | 4.93E-34 |
| *CEP55* | 2.635 | 6.99E-05 | 3.345 | 1.51E-11 | 4.087 | 3.02E-34 |
| *SFN* | 2.701 | 1.99E-03 | 2.028 | 1.31E-11 | 5.811 | 7.88E-25 |
| *CCNB2* | 2.816 | 4.95E-06 | 2.463 | 1.64E-09 | 3.892 | 8.18E-40 |
| *PTTG1* | 2.878 | 1.57E-05 | 2.050 | 3.53E-11 | 4.059 | 1.24E-33 |
| *RRM2* | 2.981 | 1.50E-04 | 3.166 | 2.16E-12 | 4.330 | 1.50E-30 |
| *PBK* | 3.218 | 1.20E-05 | 2.689 | 6.05E-09 | 4.094 | 2.24E-27 |
| *MMP12* | 3.591 | 1.77E-03 | 3.044 | 2.68E-06 | 3.723 | 4.17E-10 |

*EC: Endometrial Cancer; FC: Flod Change*
