# Supplementary figures and images for: TPX2 Serves as a Cancer Susceptibility Gene and Is Closely Associated with the Poor Prognosis of Endometrial Cancer
Source: Genet Res (Camb). 2022 Mar 16;2022:5401106. doi: 10.1155/2022/5401106 (PMC8942693; doi:10.1155/2022/5401106)

**A****Verification group**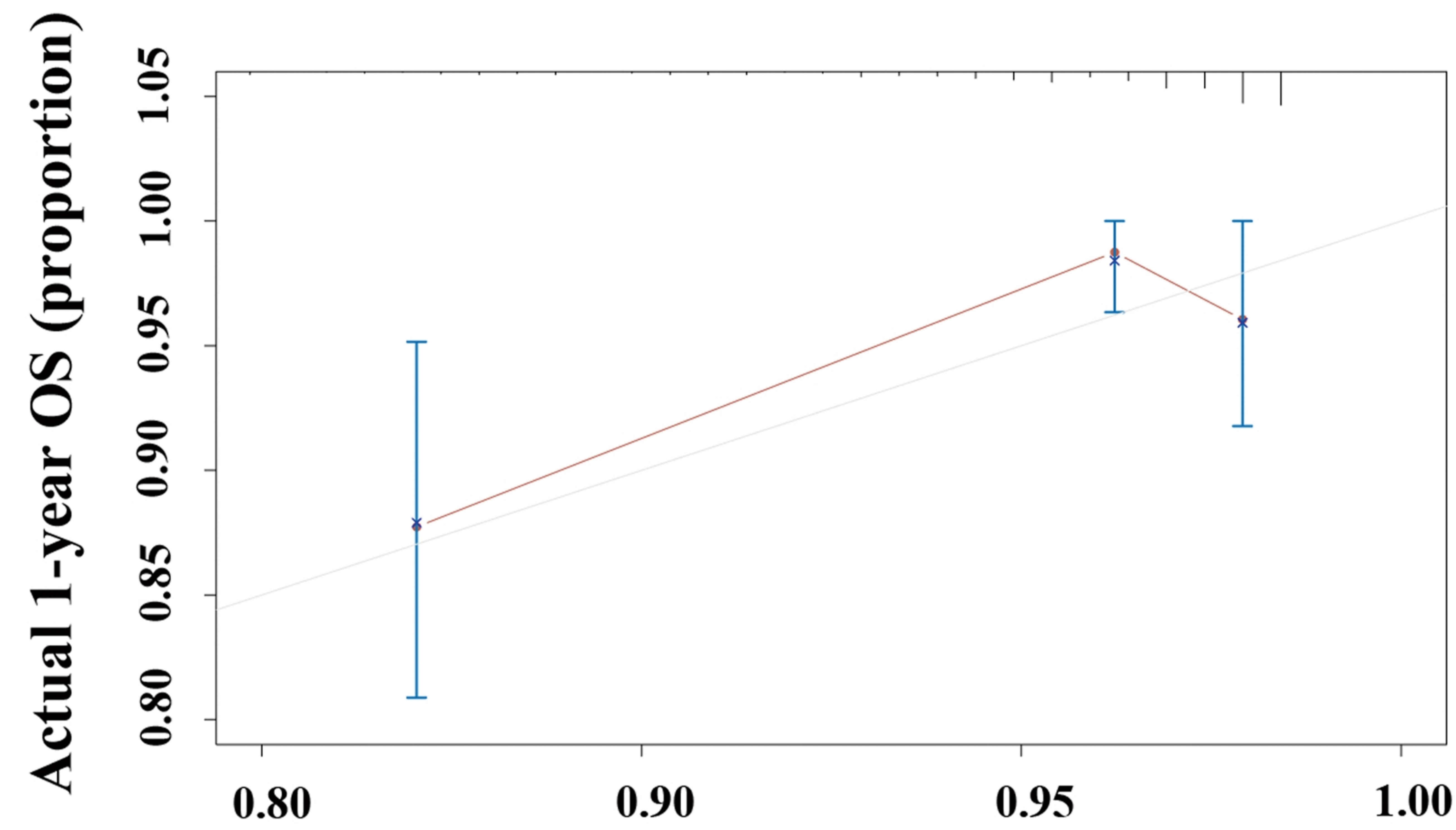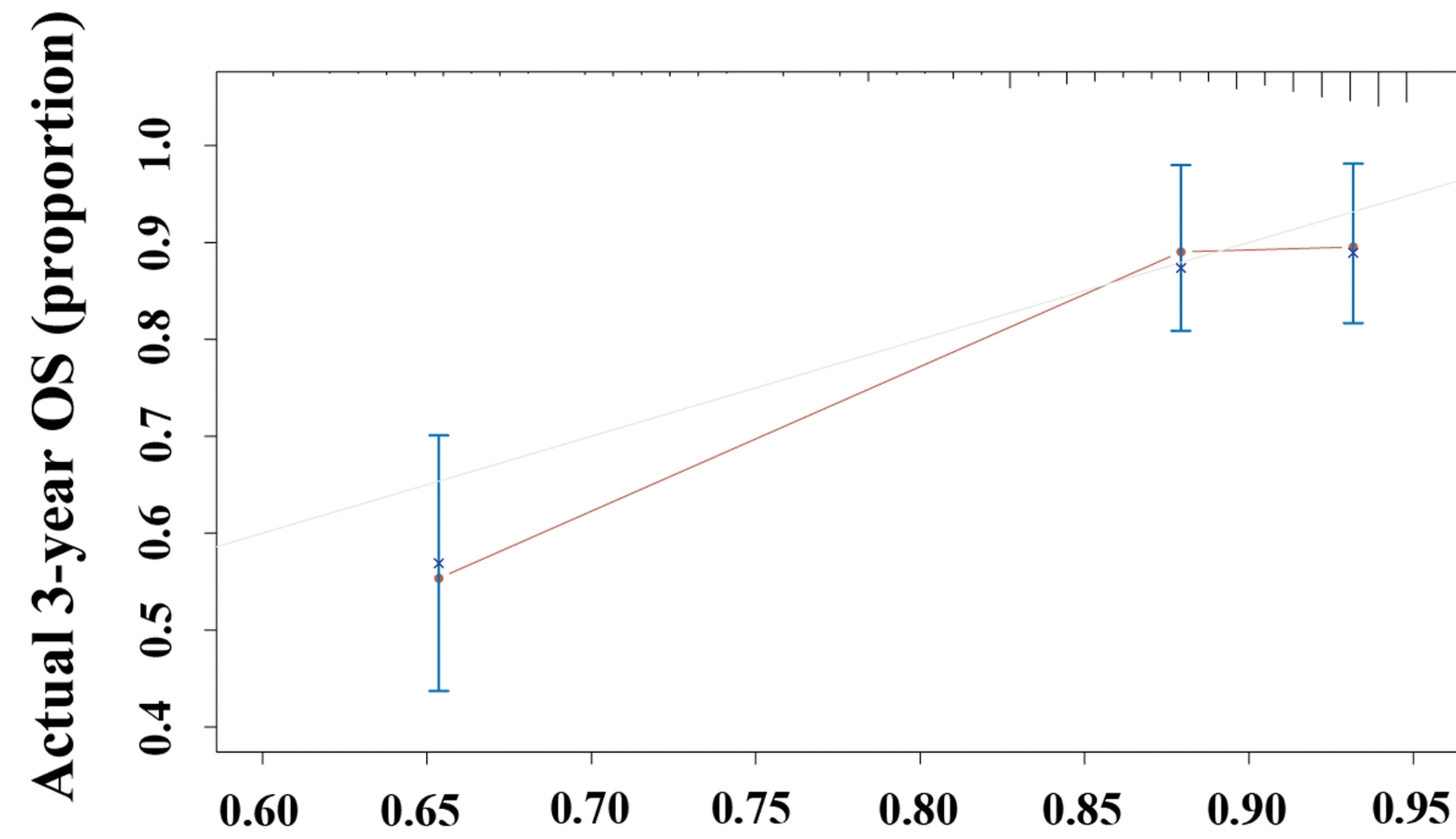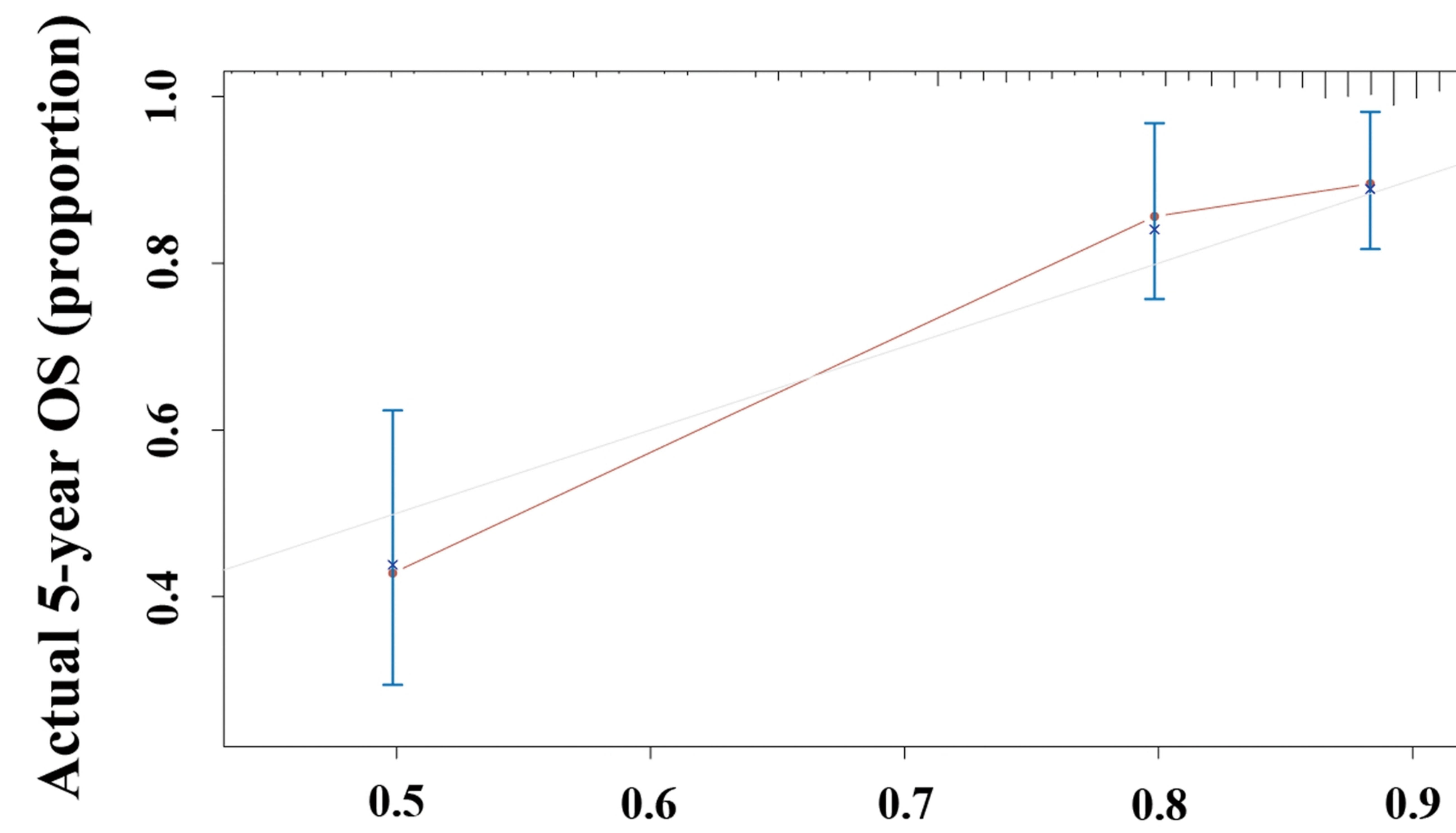**B****Combination group**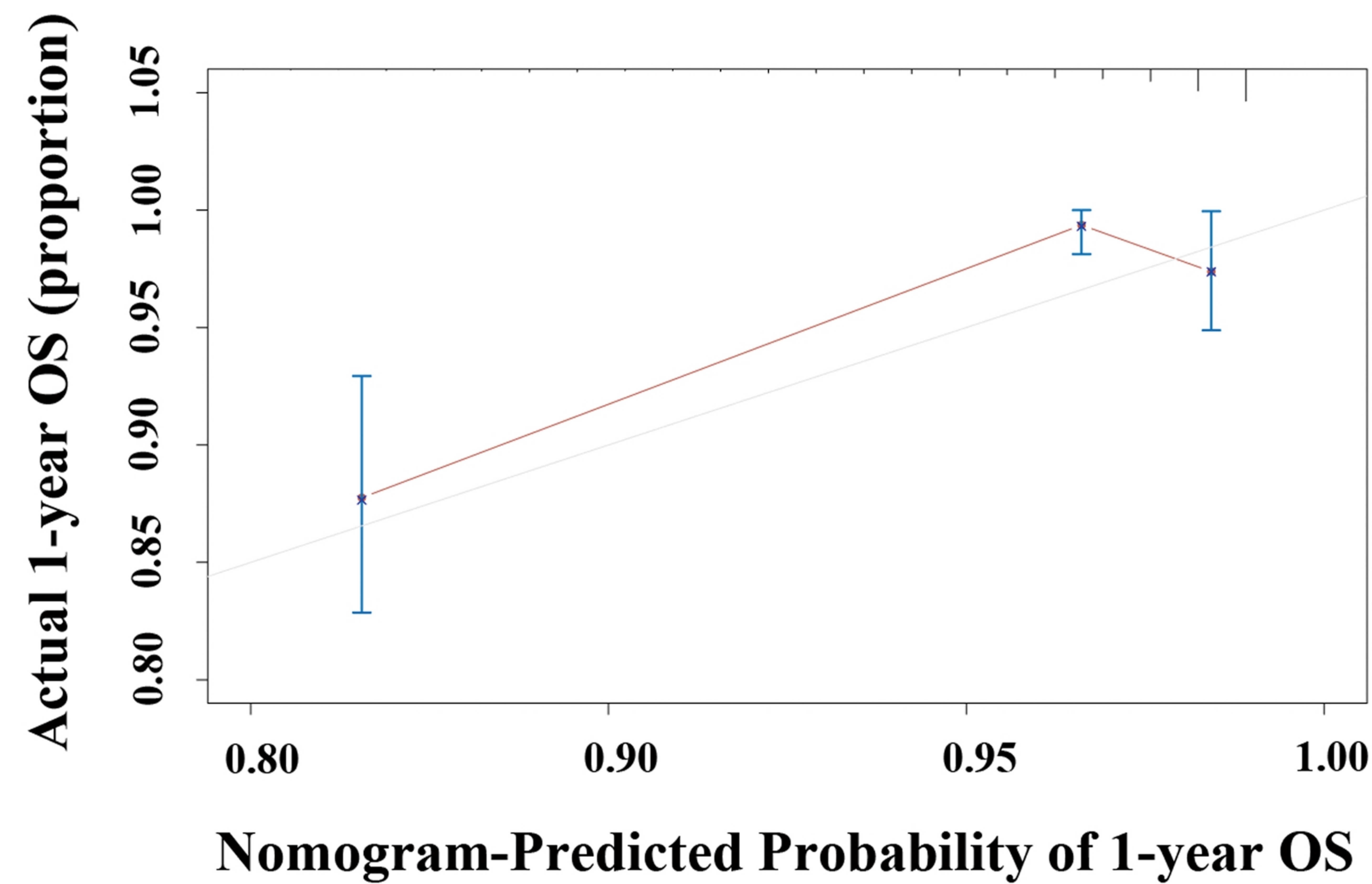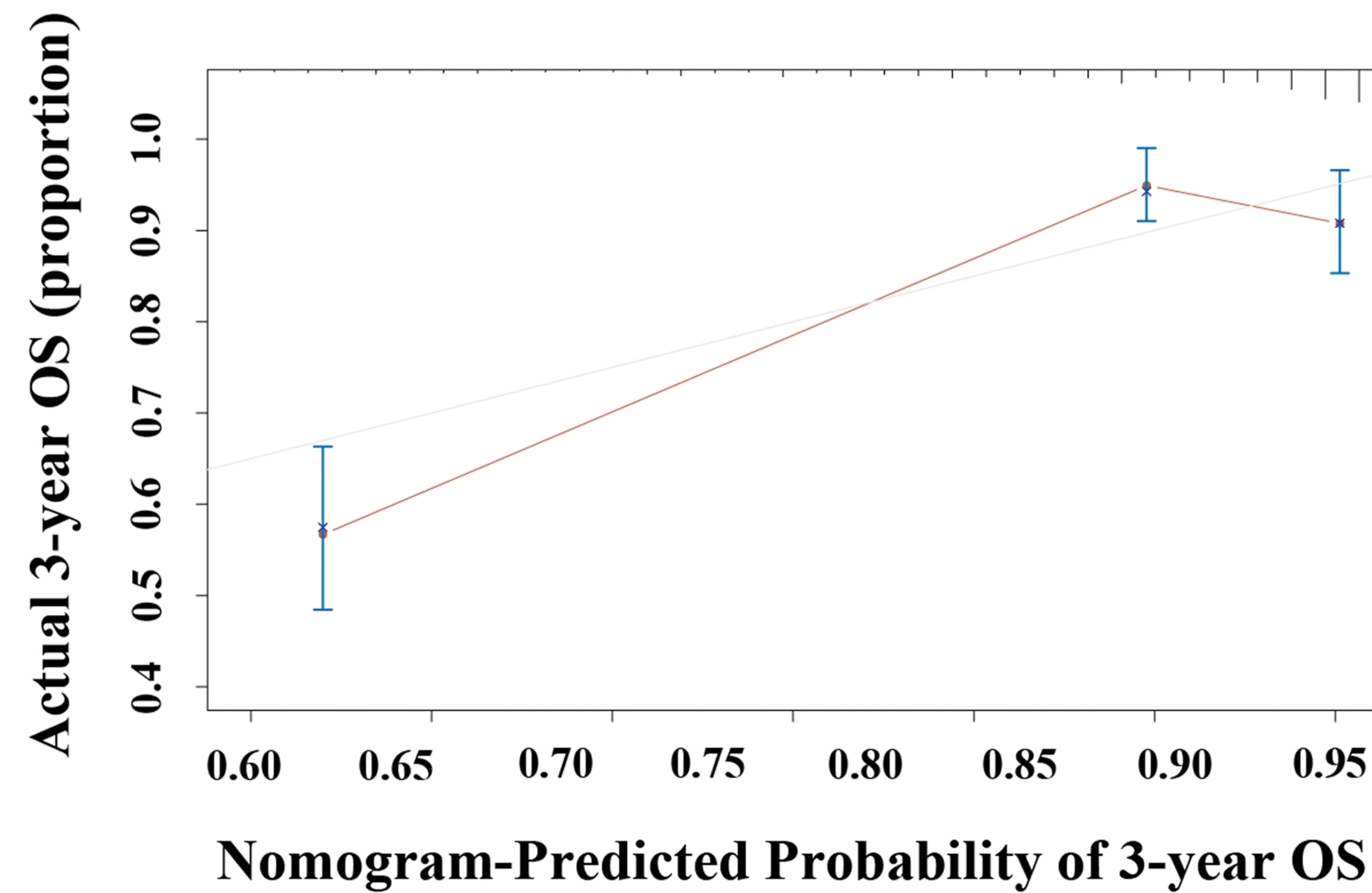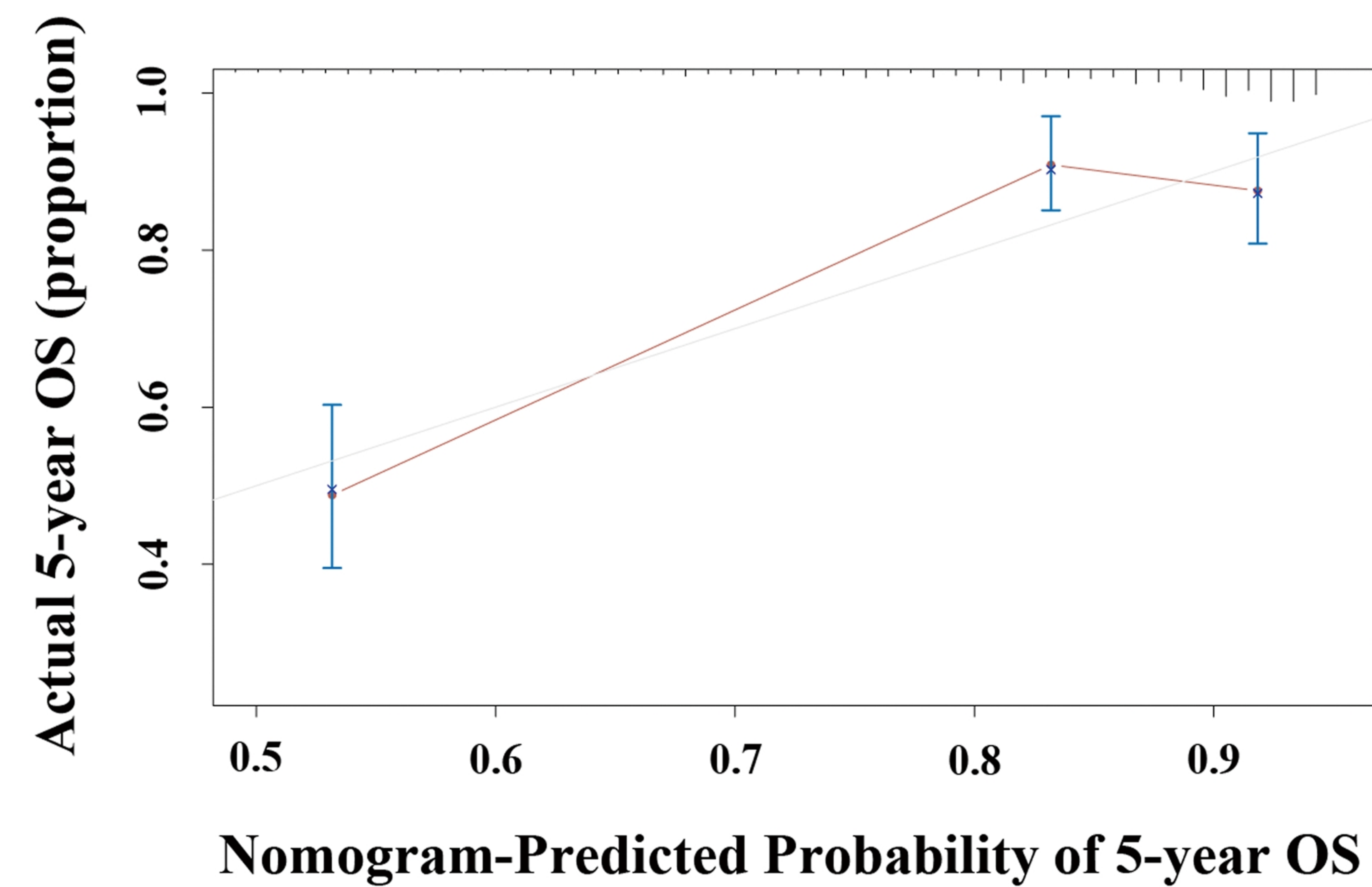

Supplement: Supplementary Materials — Supplementary Table 1: clinical features of EC patients in the training and verification groups. Supplementary Table 2: differentially expressed genes. Supplementary Figure 1: Kaplan–Meier survival curves and X-tile plots demonstrated that the optimal cutoff age was 77 years. Supplementary Figure 2: the effect of TPX2 copy number gain on OS in EC patients stratified by age (A), tumor grade (B), and histological type (C). OS: overall survival; EC: endometrial cancer. Supplementary Figure 3: the gene sets that showed enrichment in the group with low levels of TPX2 expression. Supplementary Figure 4: calibration plots of the nomogram. (A) Calibration plots for the nomogram created from the verification group predicting 1-year OS (n = 218), 3-year OS (n = 89), and 5-year OS (n = 49). (B) Calibration plots for the nomogram for the combination group predicting 1-year OS (n = 435), 3-year OS (n = 188), and 5-year OS (n = 103). The reference line represents a perfect match between the predicted and actual survival probabilities. OS: overall survival. [file 5401106.f1.zip › 5401106.f1/Supplementary Figure 4.pdf]
